# Viral Infections in Type 2 Diabetes: A Dangerous Liaison

**DOI:** 10.3390/v17091150

**Published:** 2025-08-22

**Authors:** Azizul Haque, Anudeep B. Pant

**Affiliations:** 1 One Medical Center Drive, Department of Microbiology and Immunology, Geisel School of Medicine at Dartmouth, Lebanon, NH 03756, USA; 2 New Orleans East Hospital, 5620 Read Blvd, New Orleans, LA 70127, USA; anudeep.pant1@gmail.com

**Keywords:** type 2 diabetes, viral infections, glucose homeostasis, viral proteins, immune dysregulation, inflammation, cytokines, gut microbiome

## Abstract

Type 2 diabetes mellitus (T2DM) is increasing in incidence in many parts of the world and is becoming an important global health threat. T2DM results from a disturbance in glucose metabolism and is triggered by a combination of genetic and environmental factors. In regions where diabetes is prevalent, viral infections are also common; both conditions can contribute to increased blood sugar levels. We hypothesize that under these conditions, viral infections could accelerate many of the complications of T2DM in predisposed individuals. The high glucose levels may negatively impact blood vessel structure, white blood cell function, and infection-fighting proteins, which may weaken the immune response and, in turn, increase the frequency of viral infections in diabetic patients. Furthermore, viruses can stimulate an immune response, which induces inflammation and cytokine secretion. This perspective article postulates the existence of an axis between T2DM and viral infections and highlights the mechanistic aspects underlying their connection. A better understanding of the mechanisms between viral infections and blood sugar is likely to reveal new therapeutic avenues for the treatment and management of these diseases.

## 1. Introduction

It is estimated that about 830 million people worldwide have diabetes, and cases are on the rise globally [1]. According to the World Health Organization (WHO), the majority of people with diabetes are living in low- and middle-income countries, and more than half are not receiving treatment. There is inadequate screening for diabetes in many low-income countries. Therefore, the actual number of diabetic patients is likely to be higher [2].

There are two predominant subtypes of diabetes mellitus (DM): type 1 diabetes mellitus (T1DM) and type 2 (T2DM). Both types of DM are generally considered to be prompted by the combined effect of genetic and environmental factors. T1DM results from autoimmune destruction of insulin-producing β-cells [3]. T2DM is characterized by hyperglycemia, resulting from the inability of pancreatic β-cells to produce sufficient insulin (a hormone) and the failure of cells in muscle, fat, and the liver to take in and use enough sugar, ultimately leading to peripheral insulin resistance [4]. This is also known as impaired insulin sensitivity. Insulin is essential to transport glucose from the blood into cells to be used for energy [5].

This perspective paper focuses on T2DM, which accounts for approximately 95% of cases globally [6]. In many places where diabetes is prevalent, viral infections are common, though not all diabetic individuals necessarily harbor active viral infections. We propose that viral infections, if not etiological, could trigger many of the complications of T2DM in predisposed individuals. During the coronavirus disease 2019 (COVID-19) pandemic, researchers found that the pathophysiology of hyperglycemia in diabetics was made worse by severe acute respiratory syndrome coronavirus 2 (SARS-CoV-2) infections [7]. This has sparked interest in research exploring how viral infections could contribute to augmenting blood sugar levels. While the role of some viruses has been widely studied in T1DM [8], the viral mechanisms causing T2DM have not been sufficiently investigated. Data on the reciprocity between viruses and T2DM are limited, and further studies are warranted in this field. We have examined the relationship between viruses and the development of T2DM, including known diabetogenic viruses and their mechanisms based on the recent literature on immune–endocrine interactions in the context of viral infections. It is a vast subject because of the number of viruses found in diabetic patients that may cause latent or active infections. Therefore, we will concentrate on some of the viral infections that are suspected to have a marked impact on the development of T2DM.

## 2. Viral Infections in the Context of Type 2 Diabetes

**Figure 1:**
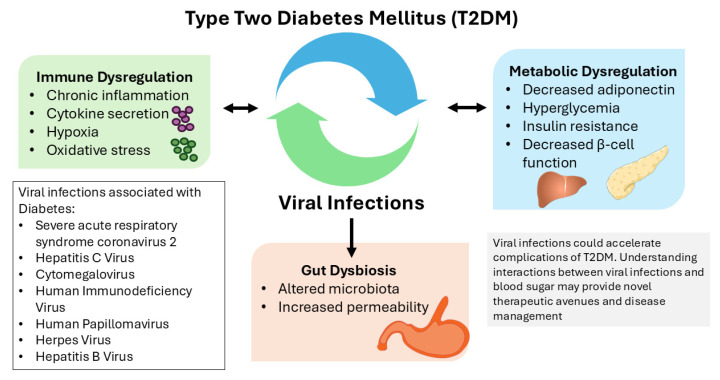
Bidirectional interactions of type 2 diabetes and viral infection. This figure illustrates the reciprocal relationship between diabetes and viral infections. Viruses can promote diabetes through chronic inflammation, gut microbiome disruption, and oxidative stress. Conversely, metabolic disruption characteristic of diabetes, such as insulin resistance or impaired β-cell function, can drive the pathogenesis of viral infections.

Among the reported viral infections associated with diabetes in humans are coronavirus 2, hepatitis C virus, cytomegalovirus, human immunodeficiency virus, human papillomavirus, herpes simplex virus, and hepatitis B virus (Figure 1). The mechanisms by which viruses induce T2DM appear to be complex, involving multiple pathways of interaction. This section covers the recent literature to illuminate the reciprocity of viral infections and T2DM.

### 2.1. The Role of Inflammatory Mediators in Virus-Induced Diabetes

In response to viral infections, the immune system produces inflammatory mediators such as cytokines and chemokines to facilitate viral clearance (Table 1). The role of cytokines and chemokines, such as TNF-α, IL-1β, IL-6, etc., in T2DM has not been sufficiently defined and investigated. These inflammatory mediators have been connected to the impairment of insulin signaling pathways and glucose homeostasis [9,10]. Tumor necrosis factor α (TNFα), a potent inflammatory cytokine, promotes insulin resistance through the downregulation of key genes required for normal insulin function [11].

Inflammation is also closely linked to hypoxia, especially in the context of viral infections and metabolic disorders [12]. Both diabetes and viral infections can cause oxidative stress through the increased production of reactive oxygen species (ROS) (Figure 1). The damage incurred by ROS may lead to beta-cell dysfunction and apoptosis, which further impacts insulin secretion [13]. Oxidative stress also contributes to insulin resistance by weakening the ability of insulin to assist cellular glucose intake [14]. An augmented synthesis of ROS may activate inhibitory pathways such as hexosamine pathways with glucose oxidation and the formation of advanced glycation end-products (AGEs) and PKCβ1/2 kinase [15].

Viral infections can also trigger physiological changes in metabolism. In diabetes, the dysregulation of metabolic processes has been highlighted as an underlying cause for the increased susceptibility to viruses [16]. Additionally, virus-induced alterations to metabolic processes have been linked to insulin resistance [17]. Clearly, there is a complex, bidirectional, and multifactorial interplay between viral infection and diabetes (Figure 1).

### 2.2. SARS-CoV-2

The presence of diabetes increases the likelihood of developing complications from COVID-19; conversely, the virus may also promote the development of diabetes [18]. Data suggest that SARS-CoV-2 may disrupt metabolic signals to produce hyperglycemia, which is often accompanied by severe disease and increased mortality [19]. More specifically, SARS-CoV-2 induced hyperglycemia by disrupting the synthesis of adiponectin, a hormone that helps regulate blood sugar levels (Table 1). Hyperglycemia, a marked trait of diabetes, contributes to chronic inflammation, induces weakened immunity against infections [2], and was recognized as a critical risk factor for severe COVID-19 early in the pandemic. Patients with hyperglycemia were 9 times more likely to be susceptible to acute respiratory distress syndrome, 15 times more likely to require intubation, and 3 times more likely to face death [19]. Many reports suggest that newly diagnosed type 2 diabetes is frequent in COVID-19 hospital admissions around the world [20,21]. It is still unclear, however, whether this trend represents truly new diabetes or previously undiagnosed cases.

The inflammatory stress caused by COVID-19 infection is likely to be a leading contributor to newly diagnosed diabetes. Cytokines generated during SARS-CoV-2 infection, particularly interleukin (IL)-6 and IL-1β, may hasten the onset of metabolic changes by affecting glucose homeostasis [22] (Table 1). It is reported that the cytokines IL-6 and IL-1β collectively can cause insulin resistance [23]. These cytokines could impact the pathogenesis of T2DM through the generation of inflammation by controlling the differentiation, migration, proliferation, and cell apoptosis. Pro-inflammatory cytokines IL-6 and IL-1β can affect IRS-1 and the PI3K pathways, resulting in defective glucose uptake [24,25]. By targeting IRS-1, IL-1β is capable of impairing insulin signaling and action and could thus participate (in combination with other cytokines) in the development of insulin resistance in adipocytes. Many pro-inflammatory cytokines can also indirectly promote insulin resistance by causing the induction of inflammatory genes, which then alter glucose uptake and insulin sensitivity (7). However, most human studies have been carried out with low numbers of participants, confounding factors have not been carefully addressed, and only a few cytokines have been investigated. Further studies are warranted to better understand how these pathways are impacted during an overlapping viral infection with T2DM.

There are also several overlapping mechanisms in the pathophysiology of diabetes and COVID-19 infection [26]. Pro-inflammatory markers such as C-reactive protein, procalcitonin, and ferritin are often augmented in both COVID-19 and diabetes [27,28]. Furthermore, immune dysregulation, enhanced inflammation, and impaired beta-cell function that characterize diabetes are also present in COVID-19 disease [29,30]. SARS-CoV-2 infections may uncover previously existing diabetes by stimulating stress, hypoxic lung injury, or by damaging pancreatic cells. Steroids used in the treatment of severe or critical COVID-19 may also result in hyperglycemia [31] (Figure 1). Finally, an increased SOCS3 expression has frequently been associated with severe COVID-19 disease [23]. Of note, SOCS3 is considered a negative regulator of insulin signaling [32]. Interestingly, inflammatory stimuli may activate the expression of STAT3, a transcription factor, which was shown to also increase SOCS3 expression [33]. Further studies are warranted to better understand the interaction between these two important transcription factors in diabetic patients with viral infections.

In addition to the overlap in immune mechanisms, Golgi protein 73 (GP73) may also contribute to the reciprocal nature of DM and COVID-19. GP73 is a stress-induced secreted factor that activates gluconeogenesis, and recent studies demonstrate that this mechanism is pertinent to elevated blood glucose levels during SARS-CoV-2 infection [34] (Table 1). Furthermore, SARS-CoV-2 enters host cells through the ACE-2 receptors [35]; as the infection progresses, ACE-2 receptors are upregulated in the pancreas, liver, adipose tissue, and small intestine, making these organs prone to viral infection [36,37]. Multi-organ inflammation resulting from SARS-CoV-2 infection induces the activation of interferon regulatory factor 1 (IRF1), which may directly impair insulin receptor substrate-1 (IRS1), leading to insulin resistance [38] (Table 1).

Taken together, these observations underline the diabetogenic potential of SARS-CoV-2; however, further studies are warranted to confirm whether SARS-CoV-2 indeed causes T2DM and, if so, whether it is due to viral persistence or other immune-mediated responses [23].

### 2.3. Hepatitis C Virus (HCV)

HCV and diabetes have also been demonstrated to have a complex and bidirectional relationship. HCV modulates glucose metabolism, promoting a nutrient-rich environment that assists viral persistence [39]. HCV core protein can mediate serine phosphorylation of IRS1 to facilitate insulin resistance [40] as well as upregulate SOCS3 and SOCS7, which are known insulin inhibitors [32,41] (Table 1). Other viral proteins, such as nonstructural protein 5A (NS5A) and envelope glycoprotein 2 (E2), have been shown to affect glucose metabolism by increasing serine phosphorylation [42] and decreasing tyrosine phosphorylation [43,44] of IRS, respectively. HCV also induces IL-1β and IL-6 [45], which, as mentioned previously, are well known for inducing insulin resistance (Table 1). Monocyte chemoattractant protein-1 (MCP1), a chemokine, is elevated in HCV infection and could be involved in promoting insulin resistance [46,47].

HCV has been shown to cause mitochondrial dysfunction, which increases cellular glucose uptake and can then stimulate lipid synthesis and lipid droplet storage in infected cells [48]. Finally, HCV can directly infect pancreatic β-cells [23], resulting in the impairment of their insulin secretory capacity. This will, in turn, aid in promoting the pathogenesis of HCV-induced diabetes [49].

### 2.4. Cytomegalovirus (CMV)

CMV is a prevalent pathogen among the general population [50] and is also a type of herpesvirus (herpesvirus type 5). CMV has been implicated in an increased risk of both type 1 and type 2 diabetes [51,52]. The detection of CMV nucleic acid sequences in the pancreatic islets of T2DM patients [53] suggests that viral infections could abet T2DM pathogenesis (Figure 1). However, other studies fail to confirm a clear association between CMV and T2DM [54].

Emerging evidence adds a new layer of complexity. One study found that in women of normal weight, CMV infection is associated with a higher risk of metabolic syndrome; conversely, women with extreme obesity exhibited a more metabolically benign profile [55]. These findings suggest that CMV’s influence on metabolism may differ based on body composition and warrant additional research.

While the diabetogenic mechanisms of CMV have been broadly investigated in T1DM, the viral mechanisms causing T2DM remain elusive [23]. The relationship between CMV infection and type 2 diabetes requires further investigation, especially through longitudinal studies, as CMV infects 45–100% of people at some time during their lives [56], and diabetes cases continue to surge globally [2].

### 2.5. Human Immunodeficiency Virus (HIV)

Individuals with HIV are living longer as a result of antiretroviral therapy (ART), which has made them at risk of aging-associated comorbidities such as diabetes [57]. Interestingly, the prevalence of diabetes in adults with HIV is higher than in the general population [58,59], and both HIV infections and treatment have been implicated in glucose dysregulation [60].

Direct effects of HIV infection, such as chronic inflammation, may also raise the risk of diabetes. Augmented levels of inflammatory molecules, such as TNFa and C-reactive protein, can impact insulin resistance [11,61]. Furthermore, growth hormone deficiency and resistance, which are commonly observed in HIV patients [62], can further abet insulin resistance [63] (Table 1). While ARTs have come a long way in terms of improving clinical outcomes and survival among individuals with HIV, these same antiretrovirals have been strongly associated with metabolic dysregulation, although the interfering effects are not the same for all protease inhibitors [64]. Protease inhibitors (e.g., atazanavir, darunavir, and saquinavir) are key components of many HIV regimens but are also implicated in the development of insulin resistance [65] (Table 1). Some medications impair glucose metabolism by interfering with glucose transporter type 4 (GLUT-4) and suppressing insulin production [66]. Treatment with other drugs, such as lopinavir and ritonavir, can elevate fasting levels of triglycerides and free fatty acids [67], but indinavir promotes insulin resistance with a minimal impact on lipid metabolism [67].

Co-infections are frequently seen in HIV individuals because of immunosuppression, which can further increase the risk of diabetes [68]. Furthermore, people with HIV are likely to develop other risk factors for diabetes, including obesity, high blood pressure, and high cholesterol. Anti-diabetic medicines should be carefully selected and monitored for potential comorbidities and drug interactions with the metabolic system in diabetes patients with HIV.

### 2.6. Human Papillomavirus (HPV)

According to the WHO, cervical cancer is the fourth leading cause of cancer in women globally, and persistent HPV is the etiological cause [69,70]. Diabetes has been shown to induce immune changes that may trigger cervical cancer [71]. In fact, rather than being a passive risk factor, diabetes may be an active cofactor that facilitates HPV-related oncogenesis [72] (Figure 1). A recent cross-sectional study found that both high and low blood glucose levels increased the risk of multiple HPV infections [73]. Despite these reported associations, the mechanisms linking HPV and diabetes remain unclear, and further studies are needed in this regard.

### 2.7. Herpes Simplex Virus (HSV)

Herpesviruses are one of the most widespread viruses in humans. At least five of the nine HSVs are widespread in human populations, and among them, HSV-1 and HSV-2 (also known as HHV-1 and HHV-2) are known to cause diseases. The HSV variants cause lifelong latent infections in their hosts [74]. Studies demonstrate associations between HSV2 and diabetes and indicate that HSV may dysregulate glucose metabolism [74,75] (Figure 1). Chronic hyperglycemia dampens antiviral immunity, facilitating viral reactivation. It has been reported that individuals with HSV2 were 59% more likely to develop prediabetes than those who were seronegative [74].

### 2.8. Hepatitis B Virus (HBV)

HBV and T2DM are both major public health concerns that most reports suggest are closely linked; however, the exact mechanisms remain unclear [76]. Chronic HBV infection damages liver tissue and induces systemic inflammation, both of which contribute to the development of T2DM [77]. On the other hand, patients with T2DM also have a high susceptibility to HBV infection through percutaneous blood exposure and due to diabetes-induced immune dysfunction [78] (Figure 1). Notably, diabetes is associated with the progression of severe liver complications, such as cirrhosis, in individuals with HBV [79]. Furthermore, the burden of liver cancer attributable to T2DM among adults infected with HBV has been exhibiting a growing trend in the last three decades [80]. It has been postulated that liver complications in T2DM comorbidity can be attributed to a combination of an elevation in free fatty acids, hepatic oxidative stress, and hyperglycemia in individuals with HBV [81,82].

### 2.9. Changes to Gut Microbiota by Viral Infections That May Influence Cell Metabolism

Recent studies implicate gut microbiome composition as a risk factor for T2DM [2,83]. Microbial dysbiosis in the gut can trigger immune dysregulation, causing inflammation, oxidative stress, and, subsequently, insulin resistance [84], all of which are drivers of diabetes (Figure 1). Viral infections, such as SARS-CoV-2, have also been shown to affect the composition of the gut microbiome [85]. This points to the emergence of the gut–lung axis and suggests that SARS-CoV-2 can directly infect the gastrointestinal tract and work in conjunction with antibiotics used to treat severe disease to promote gut dysbiosis [86]. Alterations to the gut microbiome can significantly influence immune responses as well as cell metabolism [87,88]. One of the consequences may be the disruption of the lining of the gut, also known as “leaky gut”, which may allow gut bacteria to enter the bloodstream, resulting in systemic inflammation and pathogenesis [89]. Recently, it has been reported that individuals with COVID-19 are at an increased risk of developing gastrointestinal disorders within a year after infection compared with individuals without SARS-CoV-2 infection [90]. Furthermore, changes to gut microbiota have been reported in HIV, HBV, and HCV infections [91,92,93]. These changes may impact the impairment of metabolic processes, thereby further promoting the development of T2DM. Further studies are warranted to understand the programming of metabolites due to viral infections.

## 3. Conclusions

Genetics, lifestyle, and the environment are major factors impacting the incidence of T2DM. Clearly, viruses may also contribute to the triggering or acceleration of diabetes in people with underlying predisposing factors. There are likely other underlying factors that can act in combination with viral infections to trigger virus-induced diabetes (2). Whether viral infections can directly initiate/elicit the onset of T2DM remains unknown, but the evidence demonstrates that viral infections can act as an accelerator, upregulating the metabolic process for the development of diabetes. In many tropical countries, we are seeing an increase in the incidence of diabetes. Viral infections prevalent in those countries may enhance the incidence of T2DM where they overlap with T2DM. The complex and reciprocal interplay between diabetes and viral infections was observed during the COVID-19 pandemic, where SARS-CoV-2 infection increased the risk of T2DM in susceptible prediabetic individuals. Immune-mediated changes, such as cytokine secretion in response to viral infections, appear to be an important underlying cause of insulin resistance in patients with metabolic disease. Despite mounting evidence that supports the direct and indirect associations of viruses in causing diabetes, the molecular mechanisms and specific predisposing factors of virus-induced diabetes remain an open question. The systematization and summary of data would facilitate correct comprehension of the relationship between seemingly different and unrelated disease states. A better understanding of the underlying molecular pathways of their bidirectional interactions would offer important new targets for future anti-diabetic therapies and pave the way for assessing targeted public health and preventative measures.

## Figures and Tables

**Table 1:** Viral infections involved in impairment of insulin signaling pathways and glucose homeostasis.

Virus	Targets	Impacts of Treatment	Metabolic Response	Immune Response
SARS-CoV-2	● ACE-2 virus receptor expression in the pancreas ● Direct infection of the GI tract	● Steroids used in the treatment of severe or critical COVID-19 increase blood sugar	● Disrupts adiponectin hormone production ● Golgi protein 73 (stress-induced) activates glucogenesis	● Secretion of IL-6 and IL-1β cytokines, which are well known for inducing insulin resistance ● Generation of pro-inflammatory markers (C-reactive protein, procalcitonin, and ferritin expression) contributes to chronic inflammation and insulin resistance ● Increased SOCS3 and SOCS7, known insulin inhibitors ● Activation of IRF1 impairs IRS1
HCV	● Direct infection of pancreatic β-cells results in reduced insulin secretion	● Successful treatment could lead to improved glycemic control	● Increases blood sugar ● Viral core protein mediates phosphorylation of IRS1 ● Viral protein 5A and E2 affect glucose metabolism by increasing serine phosphorylation	● Stimulates the production of IL-1β and IL-6 cytokines, which are well known for inducing insulin resistance ● Induces MCP1 chemokine, promoting insulin resistance ● Increased SOCS3 and SOCS7, known insulin inhibitors
HIV	● Direct infection of the GI tract, including gut-associated lymphoid tissue and lymphocytes in the gut	● ARTs are implicated in the development of insulin resistance ● Some medications impair glucose metabolism by interfering with glucose transporter type 4 (GLUT-4)	● Dysregulates glucose ● Growth hormone deficiency abets insulin resistance	● Augmented levels of inflammatory molecules like TNFα and C-reactive protein can impact insulin resistance
